# 4-Methyl-*N*-(4-methyl­phen­yl)benzamide

**DOI:** 10.1107/S1600536809006497

**Published:** 2009-02-28

**Authors:** B. Thimme Gowda, Miroslav Tokarčík, Jozef Kožíšek, U. Chaithanya, Hartmut Fuess

**Affiliations:** a Department of Chemistry, Mangalore University, Mangalagangotri 574 199, Mangalore, India; b Faculty of Chemical and Food Technology, Slovak Technical University, Radlinského 9, SK-812 37 Bratislava, Slovak Republic; c Institute of Materials Science, Darmstadt University of Technology, Petersenstrasse 23, D-64287 Darmstadt, Germany

## Abstract

In the title compound, C_15_H_15_NO, the amide fragment has an *anti *conformation. The central amide group is tilted with respect to the benzoyl ring, forming a dihedral angle of 32.3 (5)°. The benzoyl and aniline rings make a dihedral angle of 59.6 (5)°. Mol­ecules are linked into infinite supra­molecular chains via N—H⋯O hydrogen bonds. The mol­ecule is disordered so that the aromatic rings are disposed across a twofold axis with equal occupancies.

## Related literature

For background to our study of the substituent effects on the structures of benzanilides, see: Gowda *et al.* (2003 ▶). For related structures, see Gowda *et al.* (2008**a* ▶,b* ▶, 2009 ▶).
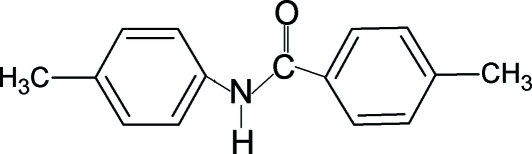


## Experimental

### 

#### Crystal data

C_15_H_15_NO
*M* *_r_* = 225.28
Monoclinic, 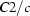

*a* = 13.3236 (5) Å
*b* = 5.3591 (2) Å
*c* = 17.3525 (6) Å
β = 92.248 (3)°
*V* = 1238.06 (8) Å^3^
*Z* = 4
Mo *K*α radiation
μ = 0.08 mm^−1^
*T* = 295 K
0.26 × 0.25 × 0.07 mm

#### Data collection

Oxford Diffraction Xcalibur System diffractometer
Absorption correction: multi-scan (*CrysAlis RED*; Oxford Diffraction, 2007 ▶) *T*_min_ = 0.984, *T*_max_ = 0.995
8166 measured reflections
1235 independent reflections
775 reflections with *I* > 2σ(*I*)
*R*_int_ = 0.029

#### Refinement

*R*[*F* ^2^ > 2σ(*F* ^2^)] = 0.037
*wR*(*F* ^2^) = 0.103
*S* = 0.99
1235 reflections
154 parameters
59 restraints
H atoms treated by a mixture of independent and constrained refinement
Δρ_max_ = 0.12 e Å^−3^
Δρ_min_ = −0.12 e Å^−3^

### 

Data collection: *CrysAlis CCD* (Oxford Diffraction, 2007 ▶); cell refinement: *CrysAlis RED* (Oxford Diffraction, 2007 ▶); data reduction: *CrysAlis RED*; program(s) used to solve structure: *SHELXS97* (Sheldrick, 2008 ▶); program(s) used to refine structure: *SHELXL97* (Sheldrick, 2008 ▶); molecular graphics: *ORTEP-3* (Farrugia, 1997 ▶) and *DIAMOND* (Brandenburg, 2002 ▶); software used to prepare material for publication: *SHELXL97*, *PLATON* (Spek, 2009 ▶) and *WinGX* (Farrugia, 1999 ▶).

## Supplementary Material

Crystal structure: contains datablocks I, global. DOI: 10.1107/S1600536809006497/tk2377sup1.cif

Structure factors: contains datablocks I. DOI: 10.1107/S1600536809006497/tk2377Isup2.hkl

Additional supplementary materials:  crystallographic information; 3D view; checkCIF report

## Figures and Tables

**Table 1:** Hydrogen-bond geometry (Å, °)

*D*—H⋯*A*	*D*—H	H⋯*A*	*D*⋯*A*	*D*—H⋯*A*
N1—H1*N*⋯O1^i^	0.883 (15)	2.416 (19)	3.202 (3)	148.3 (6)

Symmetry code: (i) 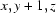.

**Fig. 1.**
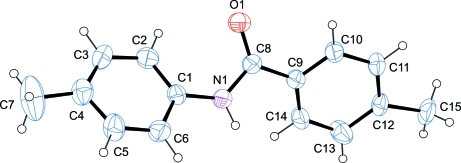
Molecular structure of (I) showing the atom labelling scheme and one orientation of the disordered molecule. Displacement ellipsoids are drawn at the 30% probability level. H atoms are represented as small spheres of arbitrary radii.

**Fig. 2.**
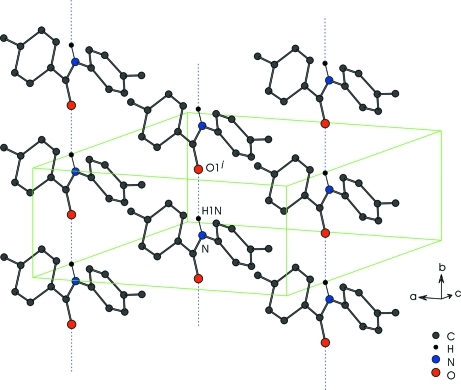
Part of the crystal structure of (I) showing supramolecular chains running along the b axis connected by N—H···Oi hydrogen bonds, shown by dashed lines. H atoms not involved in intermolecular bonding have been omitted for reasons of clarity. Symmetry operation (i): x, y +1, z.

## supplementary crystallographic information

### Comment

In the present work, as part of a study of the substituent effects on the structures of benzanilides (Gowda *et al.*, 2003; Gowda *et al.*, 2008*a*,*b*), the structure of 4-methyl-*N*- (4-methylphenyl)benzamide (I) has been determined. The amide adopts an anti-conformation (Fig. 1), similar to that observed in *N*-(4-methylphenyl)benzamide (N4MPBA) (Gowda *et al.*, 2008*a*), 4-methyl-*N*-(phenyl)benzamide (NP4MBA) (Gowda *et al.*, 2009), 2-methyl-*N*-(4-methylphenyl)benzamide (N4MP2MBA) (Gowda *et al.*, 2008*b*) and other benzanilides (Gowda *et al.*, 2003). The central amide group is tilted to the benzoyl ring at an angle of 32.3 (5)°, compared to the values of 20.7 (2)°, 33.9 (14)°, 60.0 (1)°, observed for N4MPBA,  NP4MBA and N4MP2MBA, respectively. The two aromatic rings form a dihedral angle of 59.6 (5)°, in comparison with the values in the structures cited above of 63.4 (1)°, 61.0 (1)°, and 81.4 (1)°, respectively. The molecules are linked by N—H···O hydrogen bonds (Table 1) into supramolecular chains running along the *b* axis (Fig. 2).

### Experimental

Compound (I) was prepared according to the method described by Gowda *et al.* (2003). Plate-like colourless single crystals were obtained by slow evaporation from an ethanol solution (ca. 30 ml) of (I) (0.5 g) held at room temperature.

### Refinement

H atoms attached to C atoms were placed in calculated positions and refined in the riding model approximation with C–H distances of 0.93 or 0.96 Å. The position of amide-H was refined; N-H = 0.883 (15) Å. The *U*_iso_(H) values were set at 1.2 *U*_eq_(C-aromatic, N) and 1.5 *U*_eq_(C-methyl). The structure was found to be disordered in space group C2/c. The amide-O and -H atoms lie on a 2-fold axis with the aromatic rings disordered about this axis with equal occupancies. The geometries of the rings were restrained using the SADI and FLAT commands and the anisotropic displacement parameters were restrained with the DELU command in SHELXL-97 (Sheldrick, 2008). The atoms of the aniline moiety exhibit large thermal motion which accounts for the low value of the average ring bond distance of 1.359 (6) Å.

### Crystal data

**Table:**

C_15_H_15_NO	*F*(000) = 480
*M**_r_* = 225.28	*D*_x_ = 1.209 Mg m^−^^3^
Monoclinic, *C*2/*c*	Mo *K*α radiation, λ = 0.71073 Å
Hall symbol: -C 2yc	Cell parameters from 2932 reflections
*a* = 13.3236 (5) Å	θ = 3.2–29.2°
*b* = 5.3591 (2) Å	µ = 0.08 mm^−^^1^
*c* = 17.3525 (6) Å	*T* = 295 K
β = 92.248 (3)°	Plate, colorless
*V* = 1238.06 (8) Å^3^	0.26 × 0.25 × 0.07 mm
*Z* = 4	

### Data collection

**Table:**

Oxford Diffraction Xcalibur System diffractometer	1235 independent reflections
graphite	775 reflections with *I* > 2σ(*I*)
Detector resolution: 10.434 pixels mm^-1^	*R*_int_ = 0.029
ω scans with κ offsets	θ_max_ = 26.2°, θ_min_ = 3.8°
Absorption correction: multi-scan (*CrysAlis RED*; Oxford Diffraction, 2007)	*h* = −16→16
*T*_min_ = 0.984, *T*_max_ = 0.995	*k* = −6→6
8166 measured reflections	*l* = −21→21

### Refinement

**Table:**

Refinement on *F*^2^	Primary atom site location: structure-invariant direct methods
Least-squares matrix: full	Secondary atom site location: difference Fourier map
*R*[*F*^2^ > 2σ(*F*^2^)] = 0.037	Hydrogen site location: inferred from neighbouring sites
*wR*(*F*^2^) = 0.103	H atoms treated by a mixture of independent and constrained refinement
*S* = 0.99	*w* = [exp(0.90(sinθ/λ)^2^)]/[σ^2^(*F*_o_^2^) + (0.068*P*)^2^] where *P* = 0.33333*F*_o_^2^ + 0.66667*F*_c_^2^
1235 reflections	(Δ/σ)_max_ < 0.001
154 parameters	Δρ_max_ = 0.12 e Å^−^^3^
59 restraints	Δρ_min_ = −0.12 e Å^−^^3^

### Special details

**Table:**

Geometry. All e.s.d.'s (except the e.s.d. in the dihedral angle between two l.s. planes) are estimated using the full covariance matrix. The cell e.s.d.'s are taken into account individually in the estimation of e.s.d.'s in distances, angles and torsion angles; correlations between e.s.d.'s in cell parameters are only used when they are defined by crystal symmetry. An approximate (isotropic) treatment of cell e.s.d.'s is used for estimating e.s.d.'s involving l.s. planes.
Refinement. Refinement of *F*^2^ against ALL reflections. The weighted *R*-factor *wR* and goodness of fit *S* are based on *F*^2^, conventional *R*-factors *R* are based on *F*, with *F* set to zero for negative *F*^2^. The threshold expression of *F*^2^ > σ(*F*^2^) is used only for calculating *R*-factors(gt) *etc*. and is not relevant to the choice of reflections for refinement. *R*-factors based on *F*^2^ are statistically about twice as large as those based on *F*, and *R*- factors based on ALL data will be even larger.

### Fractional atomic coordinates and isotropic or equivalent isotropic displacement parameters (Å^2^)

**Table:**

	*x*	*y*	*z*	*U*_iso_*/*U*_eq_	Occ. (<1)
O1	0.5	−0.0539 (2)	0.25	0.0805 (4)	
C8	0.52889 (16)	0.1648 (5)	0.26154 (14)	0.0581 (6)	0.5
C9	0.6171 (5)	0.2387 (10)	0.3106 (4)	0.0555 (13)	0.5
C10	0.6994 (7)	0.0833 (15)	0.3106 (7)	0.0633 (14)	0.5
H10	0.6972	−0.0616	0.2811	0.076*	0.5
C11	0.7862 (10)	0.142 (3)	0.3548 (6)	0.0640 (17)	0.5
H11	0.8413	0.0354	0.3549	0.077*	0.5
C12	0.7905 (9)	0.361 (3)	0.3989 (11)	0.061 (2)	0.5
C13	0.7076 (9)	0.522 (3)	0.3987 (9)	0.085 (3)	0.5
H13	0.7087	0.6686	0.4272	0.102*	0.5
C14	0.6229 (6)	0.4507 (13)	0.3538 (6)	0.0705 (18)	0.5
H14	0.5671	0.555	0.3533	0.085*	0.5
C15	0.8883 (6)	0.3929 (11)	0.4511 (4)	0.0850 (19)	0.5
H15A	0.8777	0.5189	0.4893	0.127*	0.5
H15B	0.9428	0.4419	0.4198	0.127*	0.5
H15C	0.9044	0.2375	0.4762	0.127*	0.5
N1	0.47296 (14)	0.3550 (4)	0.23226 (11)	0.0625 (6)	0.5
H1N	0.5	0.495 (4)	0.25	0.075*	
C1	0.3832 (5)	0.3400 (10)	0.1864 (4)	0.0567 (15)	0.5
C2	0.3155 (8)	0.1540 (16)	0.1904 (7)	0.0730 (18)	0.5
H2	0.3287	0.0205	0.2235	0.088*	0.5
C3	0.2279 (10)	0.157 (3)	0.1466 (8)	0.083 (3)	0.5
H3	0.1838	0.0245	0.1517	0.099*	0.5
C4	0.2007 (11)	0.343 (3)	0.0962 (12)	0.076 (3)	0.5
C5	0.2728 (8)	0.520 (3)	0.0939 (8)	0.081 (2)	0.5
H5	0.2613	0.6482	0.0586	0.097*	0.5
C6	0.3605 (6)	0.5322 (14)	0.1370 (6)	0.0728 (17)	0.5
H6	0.4035	0.6677	0.1329	0.087*	0.5
C7	0.1050 (8)	0.3838 (19)	0.0558 (5)	0.146 (4)	0.5
H7A	0.0578	0.2603	0.0714	0.219*	0.5
H7B	0.1134	0.3716	0.0012	0.219*	0.5
H7C	0.0805	0.547	0.068	0.219*	0.5

### Atomic displacement parameters (Å^2^)

**Table:**

	*U*^11^	*U*^22^	*U*^33^	*U*^12^	*U*^13^	*U*^23^
O1	0.0817 (8)	0.0553 (7)	0.1022 (9)	0	−0.0262 (7)	0
C8	0.0577 (18)	0.0537 (13)	0.0624 (16)	−0.0023 (10)	−0.0047 (12)	−0.0014 (11)
C9	0.0529 (18)	0.053 (3)	0.0602 (18)	0.001 (2)	−0.0042 (13)	−0.004 (2)
C10	0.052 (2)	0.063 (4)	0.074 (2)	0.004 (2)	−0.0032 (17)	−0.016 (3)
C11	0.046 (3)	0.078 (4)	0.067 (3)	0.003 (3)	−0.0077 (19)	−0.001 (2)
C12	0.045 (3)	0.077 (4)	0.058 (4)	−0.005 (2)	−0.016 (2)	0.000 (3)
C13	0.087 (5)	0.082 (4)	0.084 (4)	0.024 (4)	−0.023 (3)	−0.008 (3)
C14	0.056 (2)	0.070 (5)	0.084 (4)	0.011 (3)	−0.019 (2)	−0.004 (4)
C15	0.052 (2)	0.104 (3)	0.097 (5)	−0.017 (2)	−0.021 (2)	0.001 (3)
N1	0.0602 (15)	0.0521 (11)	0.0736 (15)	−0.0012 (9)	−0.0166 (9)	−0.0010 (9)
C1	0.0564 (17)	0.057 (4)	0.056 (2)	0.004 (3)	−0.0120 (15)	0.004 (3)
C2	0.075 (5)	0.064 (4)	0.080 (3)	0.000 (3)	−0.003 (3)	0.024 (3)
C3	0.058 (5)	0.085 (4)	0.104 (5)	−0.017 (3)	−0.004 (3)	0.002 (3)
C4	0.069 (5)	0.096 (5)	0.063 (4)	0.003 (3)	−0.007 (4)	−0.015 (3)
C5	0.073 (4)	0.104 (5)	0.063 (3)	0.003 (3)	−0.017 (3)	0.016 (3)
C6	0.074 (4)	0.068 (4)	0.076 (3)	−0.007 (3)	−0.007 (3)	0.013 (3)
C7	0.100 (5)	0.274 (11)	0.061 (3)	0.040 (5)	−0.025 (3)	0.001 (4)

### Geometric parameters (Å, °)

**Table:**

O1—C8	1.247 (3)	N1—N1^i^	0.929 (3)
O1—C8^i^	1.247 (3)	N1—C1	1.413 (6)
C8—C8^i^	0.854 (4)	N1—H1N	0.883 (15)
C8—C9	1.478 (7)	C1—C2	1.348 (5)
C9—C14	1.362 (6)	C1—C6	1.366 (5)
C9—C10	1.377 (5)	C2—C3	1.368 (7)
C10—C11	1.398 (7)	C2—H2	0.93
C10—H10	0.93	C3—C4	1.363 (7)
C11—C12	1.402 (7)	C3—H3	0.93
C11—H11	0.93	C4—C5	1.352 (7)
C12—C13	1.400 (6)	C4—C7	1.447 (14)
C12—C15	1.567 (11)	C5—C6	1.363 (8)
C13—C14	1.398 (10)	C5—H5	0.93
C13—H13	0.93	C6—H6	0.93
C14—H14	0.93	C7—H7A	0.96
C15—H15A	0.96	C7—H7B	0.96
C15—H15B	0.96	C7—H7C	0.96
C15—H15C	0.96		
			
C8^i^—C8—C9	162.2 (4)	C2—C1—N1	124.5 (6)
O1—C8—C9	125.3 (3)	C6—C1—N1	118.2 (6)
C14—C9—C10	118.4 (6)	C1—C2—C3	121.3 (9)
C14—C9—C8	124.6 (6)	C1—C2—H2	119.3
C10—C9—C8	117.0 (6)	C3—C2—H2	119.3
C9—C10—C11	120.4 (9)	C4—C3—C2	124.5 (12)
C9—C10—H10	119.8	C4—C3—H3	117.8
C11—C10—H10	119.8	C2—C3—H3	117.8
C10—C11—C12	120.2 (11)	C5—C4—C3	111.0 (12)
C10—C11—H11	119.9	C5—C4—C7	119.5 (11)
C12—C11—H11	119.9	C3—C4—C7	129.0 (10)
C13—C12—C11	119.9 (10)	C4—C5—C6	127.8 (13)
C13—C12—C15	124.9 (9)	C4—C5—H5	116.1
C11—C12—C15	114.9 (8)	C6—C5—H5	116.1
C14—C13—C12	116.9 (10)	C5—C6—C1	118.1 (10)
C14—C13—H13	121.5	C5—C6—H6	121
C12—C13—H13	121.5	C1—C6—H6	121
C9—C14—C13	124.1 (8)	C4—C7—H7A	109.5
C9—C14—H14	117.9	C4—C7—H7B	109.5
C13—C14—H14	117.9	H7A—C7—H7B	109.5
N1^i^—N1—C1	172.2 (4)	C4—C7—H7C	109.5
N1^i^—N1—H1N	58.3 (7)	H7A—C7—H7C	109.5
C1—N1—H1N	124.7 (6)	H7B—C7—H7C	109.5
C2—C1—C6	117.2 (6)		
			
C8^i^—O1—C8—C9	169.7 (6)	C8—C9—C14—C13	−178.6 (11)
C8^i^—C8—C9—C14	1.0 (19)	C12—C13—C14—C9	0(2)
O1—C8—C9—C14	−145.7 (6)	C6—C1—C2—C3	0.1 (17)
C8^i^—C8—C9—C10	−177.5 (15)	N1—C1—C2—C3	176.9 (10)
O1—C8—C9—C10	35.8 (9)	C1—C2—C3—C4	0(3)
C14—C9—C10—C11	0.6 (15)	C2—C3—C4—C5	1(3)
C8—C9—C10—C11	179.2 (9)	C2—C3—C4—C7	−170.1 (17)
C9—C10—C11—C12	−1(2)	C3—C4—C5—C6	−3(3)
C10—C11—C12—C13	0(3)	C7—C4—C5—C6	169.1 (16)
C10—C11—C12—C15	175.0 (12)	C4—C5—C6—C1	4(3)
C11—C12—C13—C14	0(3)	C2—C1—C6—C5	−1.7 (15)
C15—C12—C13—C14	−174.0 (15)	N1—C1—C6—C5	−178.8 (10)
C10—C9—C14—C13	−0.2 (16)		

Symmetry codes: (i) −*x*+1, *y*, −*z*+1/2.

### Hydrogen-bond geometry (Å, °)

**Table:**

*D*—H···*A*	*D*—H	H···*A*	*D*···*A*	*D*—H···*A*
N1—H1N···O1^ii^	0.88 (2)	2.42 (2)	3.202 (3)	148 (1)

Symmetry codes: (ii) *x*, *y*+1, *z*.
